# ‘I Do Often Feel Like We Were Given a Magic Wand’ – Children's and Families’ Experiences of Allied Health Services for Developmental and Disability Needs in Rural and/or Remote South Australia

**DOI:** 10.1111/hex.70820

**Published:** 2026-08-14

**Authors:** Georgia Gosse, Saravana Kumar, Helen Banwell, Joshua Pate

**Affiliations:** ^1^ Allied Health and Human Performance, Innovation Implementation and Clinical Translation in Health Adelaide University North Terrace SA Australia; ^2^ Graduate School of Health/School of Health University of Technology Sydney Broadway Ultimo NSW Australia

**Keywords:** children, developmental delay, disability, models of care, paediatric, qualitative research, strengths‐based

## Abstract

**Background:**

Children in rural and remote areas are increasingly developmentally vulnerable compared to metropolitan peers. Allied health professionals are positioned to provide services to support children with developmental and disability needs but face barriers to delivering services in rural and remote areas. Research has highlighted the need to understand context, for quality care delivery; however, critical gaps remain in understanding assets within our communities.

**Objective:**

The aim of this study was to explore parents/carers and children's experiences receiving services for disability and developmental needs in rural and remote South Australia.

**Design:**

Qualitative appreciative inquiry methodology using semi‐structured interviews and.

**Setting and Participants:**

Participants were purposefully recruited via snowball sampling through allied health professionals and included parents/carers and children receiving allied health services for developmental and/or disability needs.

**Results:**

Thirteen parents, one carer and eight children participated in interviews. Findings were inductively structured into a socio‐ecological framework. Themes included ‘the importance of having fun’, ‘therapy makes us grow’, ‘my child and therapist are parts of the community’, ‘consistent, knowledgeable and caring therapists’, ‘flexible environments and health services’ and ‘accessing funding models and the healthcare system’.

**Discussion:**

These findings tell us that the value of ‘fun’ and community within health services should not be overlooked. Parents/carers value therapists’ individual qualities, seeing their children grow and being supported to understand the healthcare system.

**Conclusions:**

Children's and parents/carers perspectives should be centred so communities are positioned to drive sustainable and practical improvements in allied health service delivery.

**Patient of Public Contribution:**

This study sought experiences from parents/carers and children with developmental or disability needs in rural and remote South Australia. The lead author is an allied health professional who has been working with this population. All participants were sent the results for feedback. The findings will inform stakeholders developing models of care for delivering allied health services to this population.

## 1 Introduction

Healthcare in rural and remote Australia faces challenges related to geographical isolation, most notably, restricted accessibility [1]. While rural and remote Australia boasts the benefits of increased sense of connectedness and housing opportunities, these areas experience higher levels of poor health outcomes and significant challenges with accessing appropriate healthcare [1, 2, 3]. South Australia is one of the least densely populated states in the world with 24.3% living outside of metropolitan areas [4]. Children in Australia are becoming increasingly developmentally vulnerable [5]. This relates to children retaining skills below age expectations in areas of communication skills, language and cognitive skills, emotional maturity, physical health and wellbeing, and general knowledge [5]. In very remote South Australia, 43.5% of children are developmentally vulnerable, compared to 24% in South Australia broadly [5]. Early intervention for developmental delay is associated with improved social, health and economic consequences. Within Australia, the economic cost of late intervention of childhood challenges has been attributed to $22.3 billion (AUD) in 2024 [6]. This includes costs related to healthcare later in life [6]. In South Australia, health and disability services are funded and delivered through separate systems. Children receive free developmental checks via government‐funded Medicare. Allied health support for children with a formal developmental diagnosis/disability, by contrast, is predominantly funded through the National Disability Insurance Scheme (NDIS), a federal social insurance that supports individuals with lifelong diagnoses [7].

A range of allied health professionals (AHPs), such as physiotherapists, occupational therapists, and speech therapists, provide services to children and families to address these developmental challenges [8, 9, 10]. Children require a more dedicated service due to the complex nature of development, and the requirement for family‐centred services, often making it more challenging to find the right professional [11]. These services ensure children can participate in their community, and without these services, they are increasingly vulnerable [12]. Additionally, children have historically been excluded from research due to pragmatic challenges associated with their inclusion, such as their ability to comprehend concepts [13, 14, 15, 16]. However, it is supported that children can comprehend concepts relating to their healthcare [13, 17]. The UN Convention on the Rights of the Child acknowledges that children have a right to express opinions and have their views heard [18]. Including children in research allows us to capture these experiences of healthcare use, allowing for better engagement, improved health and health service outcomes and preventing exclusion [14]. Considering adults’ perspectives only risks misrepresenting children and fails to capture the true end‐user of the children's services [16].

To deliver these allied health services to areas experiencing accessibility challenges, various models of care have been identified for use [11, 19]. While there is heterogeneity in the styles of models of care delivered, all models of care agree that the needs and assets of the communities where services are delivered are most important [20]. This identifies that including communities and end‐users (including children) to shape decisions before a service is implemented offers the best outcomes [20].

A strengths‐based approach to investigating healthcare and community collaborations offers high‐value outcomes [21], but it is commonly underused. Strengths‐based approaches, such as appreciative inquiry methodology, focus on where there is currently success in service delivery and can shift what resources we might focus on when trying to make change [21, 22]. These approaches can do this whilst acknowledging the inherent challenges or prejudice faced in populations, such as children in rural and remote areas [23]. There is already extensive research highlighting the barriers and challenges in rural and remote areas, creating a deficit‐based narrative surrounding healthcare in these areas [2, 24, 25]. Therefore, a strengths‐based approach to undertake research in this area has the potential to not only shift this narrative but also uncover assets, goals, and new possibilities within communities.

The aim of this study was to use a strengths‐based approach to understand the experience of parents/carers and children receiving allied health services for development and/or disability needs in rural and remote South Australia.

## 2 Materials and Methods

### 2.1 Study Design

We used appreciative inquiry methodology to capture the experiences of participants through a strengths‐based lens [22, 26, 27, 28]. Appreciative inquiry is a person‐focused methodology that explores assets and resources to develop new service ideas [28]. The approach involves four stages: ‘Discover’, ‘Dream’, ‘Design’ and ‘Destiny’ [22, 26]. These stages respectively focus on identifying existing strengths of services (discover), envisioning ideal possibilities (dream), determining the requirements to achieve these visions (design) and strategies to sustain and future‐proof the changes (destiny) [26]. The full methodology has been published elsewhere (manuscript under review).

A Consolidated criteria for Reporting Qualitative research (COREQ) checklist is in Supporting Information: File SA [29].

The Women's and Children's Network Human Research Ethics Committee (HREC) (Reference 042917) and the University of South Australia HREC (Reference 206707) approved this project. All six regional local health networks (South Australia) provided approval for site‐specific assessments. This study was prospectively registered on Open Science Framework in July 2024 (https://osf.io/hc7j4).

### 2.2 Sample and Setting

Parents/carers and children were recruited via snowball sampling from AHPs involved in a previous study. AHPs were provided with information to identify families that may be appropriate (as per the inclusion criteria listed below). The treating AHP had no further involvement in the interview process, reducing the potential for participants to feel they could not share openly. No information about the outcomes of the parent/carer/child interventions were shared with the AHPs. The lead research (G.G.) was not known to any participants. Parents/carers who were willing to be involved following discussions with the AHP, signed a consent‐to‐contact form.

### 2.3 Data Collection

Semi‐structured interviews allowed for in‐depth discussion and flexibility when talking with parents/carers and children [30]. Interviews were conducted (GG and alternate research team member) in person, in clinics familiar to clients, and online via Zoom™ (https://zoom.us). Children were always accompanied by a parent/carer, in these instances interviews were conducted concurrently. This meant that a child would be asked a question (as per their question guide) and then the parent may be asked a question while the child drew or played. Parents/carers were advised to not encourage any particular answers from their child and that there was no incorrect way for them to respond to the questions, This was done as per ethical requirements.

**Figure 1:**
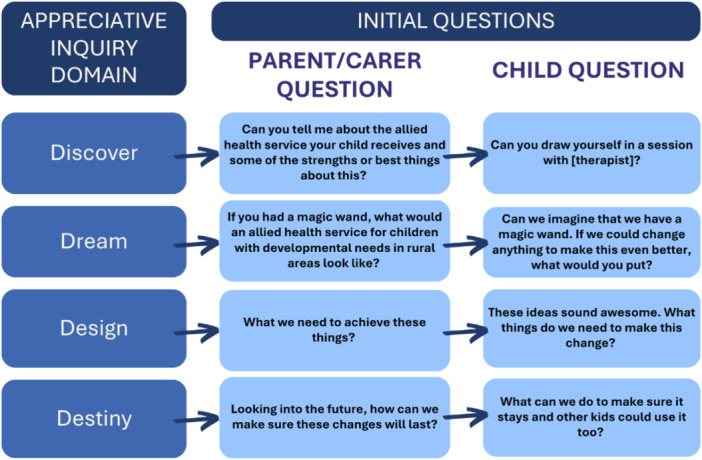
Basic question guide for parents/carers and children based on appreciative inquiry domains.

Interview questions were developed via iterative discussions with three paediatric researchers. Feedback included clarifying age‐appropriate language and activities. Photovoice (drawing) and ‘small parts play’ methods were also included for the children [15, 31, 32]. A collection of drawing items and toys (e.g., constructive play and imaginative play items) were used to build rapport and to triangulate ideas [32]. All questions, photovoice and small parts play methods were piloted with two parent/child dyads to clarify wording, set up of space and practice online discussions. Piloting resulted in the room set‐up so that toys were kept away from children initially to avoid distraction, and to keep the initial focus on the drawing materials. Piloting also demonstrated that children can require several minutes to sit and draw without being asked direct questions. At the end of the interviews, parents/carers completed a demographic form for their child and themselves (see Supporting File B). Figure 1 shows a basic question guide mapped to the appreciative inquiry domains (full guide can be found in Supporting Information: File SC).

Recruitment continued until the accessible pool of eligible participants was exhausted. Rather than applying data saturation as a stopping criterion, we assessed the adequacy of the sample using the concept of information power. Given the focused aim of this study, we judged the dataset to hold sufficient information power to address the research question. Additionally, pragmatic reasons restricted the recruitment timeframe.

### 2.4 Data Analysis

Braun and Clark's thematic analysis was used as a guide for the analysis [33]. Data was also transcribed following Braun and Clark's recommendations for transcribing data [34]. GG became familiar with the data through listening to the transcripts and completing verbatim transcriptions. Twenty per cent of transcripts were checked by alternate research team members. All data was deidentified, and quotes were copied into Excel™. GG used an inductive approach to develop codes based on appreciative inquiry. The research team confirmed these codes and iteratively refined the themes. Through ongoing discussion and refinement, the themes were subsequently organised into domains aligned with the socio‐ecological framework. Informing this study through the socio‐ecological framework helps to conceptualise the findings through the dynamic interaction between the child and nested systems surrounding them. The surrounding systems included the immediate family and therapists (microsystem), community settings (mesosystem), and broader structural and policy contexts such as service funding systems (macrosystem). This framework was selected because it allows for examination of how changes occur at multiple levels, acknowledging the complexity of change in healthcare systems, particularly in rural and remote areas(SEF) [35]. This has been replicated in other research [22].

### 2.5 Rigour

Multiple strategies were used to ensure rigour across the study. Rigour is ensured through credibility, dependability, confirmability and transferability [36]. Credibility was ensured through peer debriefing and reflexivity; triangulation of data from interviews and play methods; and member checking. Dependability and confirmability occurred through a clear audit trail of decisions, supervisor feedback, and team discussion. Transferability was ensured through field notes, collection of demographic information, and the inclusion of diverse parents/carers/children.

### 2.6 Reflexivity

The research team's position and variation in skills and experiences allowed for robust discussions. GG is a physiotherapist and PhD candidate with experience working with children in rural and remote South Australia. She currently works in both metropolitan and remote paediatric settings. SK is a physiotherapist, professor and lecturer with specialist knowledge in health services research. HB has a clinical background in paediatric services as a credentialed paediatric podiatrist, is a programme director of her discipline, and a senior lecturer. JP has experience working with children as a physiotherapist and within qualitative research. He is currently a senior lecturer. Due to the background of the lead researcher (G.G.), further trust was able to be built between the participants during the interviews. This also positively influenced G.G.'s ability to engage with children and set up the interview in a child and family‐friendly way. As G.G. was not known to any participants, it is unlikely that this influenced how participants responded. The influence of different levels of experience (variation in clinical experience, paediatric experience and rural experience), allowed for robust discussion during the inductive research process. The interpretation of the results was influenced by these different experiences.

GG conducted all data collection and did not know or have a relationship with any participants. The participants were aware of the aims of the research but did not know the questions in advance. Participants were provided information about GG's background of work and stage of research at the start of the session.

### 2.7 Results

**Figure 2:**
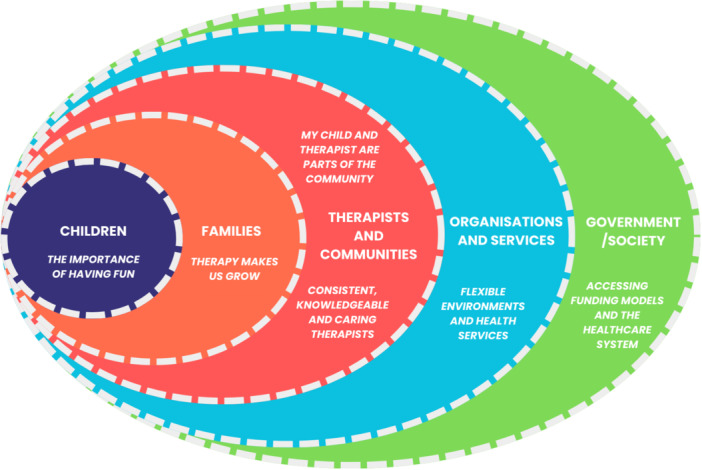
Diagrammatic representation of themes based on the socio‐ecological framework.

A total of 13 parents, one carer and eight children participated in interviews from four of the six local health networks in South Australia (Table 1). Interviews lasted between 30 and 60 min. All parents/carers and children consented to audio‐recording and capturing of pictures drawn. Parents/carers had an average age of 42, with one parent identifying as Aboriginal. No parent/carers or children identified as culturally and linguistically diverse. There was a broad range of diagnoses included, as well as varied allied health professional support. One child used eye‐gaze and non verbal communication, three children provided drawings that responded to the questions (along with verbal and non‐verbal involvement), while seven children provided verbal responses. No parents/carers or children declined to participate, however two parents/carers were lost to follow up following initial contact from researcher. Table 2 outlines the participant characteristics. Figure 2 displays the themes generated, mapped onto the domains of the socio‐ecological framework.

**Table 1:** Inclusion and exclusion criteria.

Inclusion criteria	Exclusion criteria
Parent/carer with a child who has received allied health services in the last 6 months. Living in rural or remote SA (MM 2–7)[37]^a^ Receiving services in rural or remote SA (MM 2–7) [37] Children aged 5–18. Any communication style or need^b^ [3]	Metropolitan‐based parent/carer and child. Receiving only metropolitan‐based services. Parent/carer inability to read and comprehend English.

^a^ Modified Monash Model (MM) is used as per the Australian Government Department for Health and Aged Care to define rurality based on location and population size [37]. Rural and remote South Australia are defined as per the Modified Monash Definitions.

^b^E.g., augmented and alternate communication.

**Table 2:** Participant characteristics.

Characteristic	n
**Parent/carer**	14
Female	13
Age (average)(years)	42
**Children**	8
Female	3
Age (average)(years)	8
Age (range) (years)	5–15
**Diagnoses** a	
* Attention Deficit Hyperactivity Disorder*	5
* Autism Spectrum Disorder*	5
* Delayed language skills/Dysphasia*	3
* Cerebral palsy*	2
* Low muscle tone*	2
* Anxiety*	1
* Charcot‐Marie Tooth*	1
* Chromosomal condition*	1
* Cleft palate*	1
* Down syndrome*	1
* Epilepsy*	1
* Hydrocephalus*	1
**Allied health disciplines accessed**	
* Occupational therapist*	13
* Speech pathologist*	11
* Physiotherapist*	8
* Dieticians*	5
* Exercise physiologist*	4
* Psychologist*	4
* Social worker*	4
* Audiologists*	3
* Podiatrist*	3
* Behaviour therapist*	1
**MM** b **location (location from parent/carer response)**	
* MM3*	4
* MM4*	1
* MM5*	6
* MM6*	2

^a^ Some children had multiple diagnoses reported. Diagnoses were reported individually, and not as clusters.

^b^ Modified Monash Model (MM) is used as per the Australian Government Department for Health and Aged Care to define rurality based on location and population size [37].

Throughout the results, the shortened version of ‘parent’ will be used to describe parent/carer unless the person identified solely as a carer.

**Key:** C=child, D=parent from a dyad, P=parent.

### 2.8 Domain 1: Children

#### 2.8.1 Theme 1: The Importance of Having Fun

**Figure 3:**
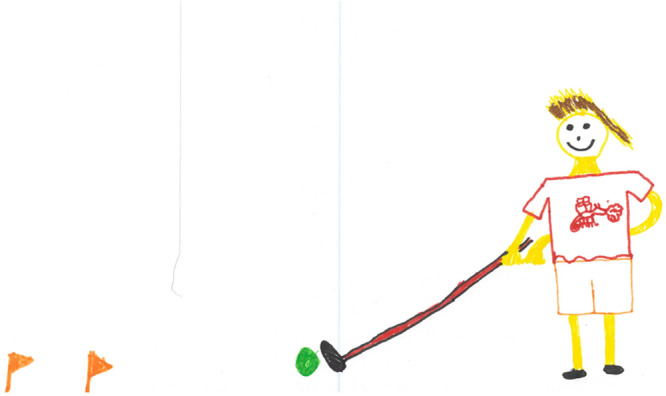
Child drawing themselves smiling and completing their favourite activity during a physio session.

All children reported ‘fun’ in allied health therapy sessions as a strength of the services and celebrated therapists’ ability to ‘be able to make them a bit fun while still like doing what I need.’ (15‐year‐old (yo) child, MM5) In drawings, such as in Figure 3, children drew the setup of obstacle courses, toys and swing sets with their faces frequently smiling.

**Figure 4:**
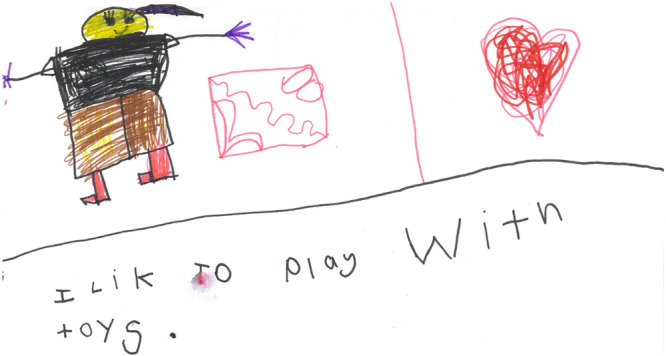
Child drawing their experience of their allied health appointment (6yo child, MM5, C6).

Using eye gaze on a picture board, a parent/carer identified the child responding ‘happy’ when asked ‘how do you feel when you see [OT]?’ Figure 4 is a drawing from a child showing them smiling with toys and her writing that she likes to play with toys.

Children were also asked what they would tell a friend who was nervous about attending an allied health session.

‘don't be scared, she has lots of games (6yo girl, MM5, C6)’ and ‘they don't hurt you.’ (8yo boy, MM5, C7)

The experience of children was also shared from their parents’ point of view. Frequently, parents discussed the strength of the relationship between their child and the therapist.

‘my kid is extremely shy and suffers from anxiety with people that he doesn't know. He's been able to build a lot of rapport with his therapist.’ (Mother, MM6, P1)

Parents also perceived their children having fun as a strength of the sessions, with reports such as, ‘every time I've gone to pick her up with [OT]. I can hear her laughing, so she loves it.’ (Father, MM5, D3)

When children were asked how we could make ‘Dream’ services for them, they reported ‘more fun! (7yo boy, MM3, C8)’ and ‘kind of just make all of them into like something fun, while still easily like doing what the purpose of the session is.’ (15yo girl, MM5, C4) It was also suggested for therapists to ‘be more entertaining’ (8yo boy, MM5, C7) and therapists to have ‘toys like what you can play with, and…talk to them [therapists] and stuff.’ (8yo child, MM5, C7)

### 2.9 Domain 2: Parents/Family

#### 2.9.1 Theme 2: Therapy Makes Us Grow

Parents reported seeing their child grow as a strength of receiving the services and were often surprised at the goals achieved.

‘My favourite things about sessions would be watching my son engage with them and achieve something.’ (Mother, MM6, P1)

‘to see the achievement where she's grown has blown me away so. And that's in 3 years (Father, MM5, D3)’.

It was reported that seeing their children grow also meant seeing them participate more.

‘being in a rural area and providing those services is just so important for the community; it made a difference between [child] not being able to run, and not being engaged in his community.’ (Mother, MM5, P4)

Seeing growth also extended to the parents/carers feeling supported in their parenting and how they could continue to support their child.

‘It was what it gave, what accessing these services, what it meant for myself and my partner and the tools…that it gave us to be able to support [child] best.’ (Mother, MM5, P2)

One parent/carer shared that initially, their early experience of the healthcare system was not positive and ‘that experience…was really bad. I felt really alone. I felt like I had this baby that I didn't know how to feed or how to help.’ (Mother, MM5, D5) Another family reported this later in their experience.

‘we feel like we're floundering. We feel like we're drowning…I just think there's a real lack of support for both…the child, but also the family supporting that child.’ (Grandmother, MM3, D8)

A need for early intervention services was reported as important to families with parent/carers, acknowledging ‘those supports and connections in those early years are vital to being able to help on the right path.’ (Mother, MM5, D2) Ongoing support was also requested ‘to know that we're doing the right thing.’ (Grandmother, MM3, D8) However, some parents reported that the engagement in ongoing support was not consistent between families.

‘It's really an individual thing…you can help and help people to be blue in the face, and you'll get nowhere. So for the ones that want and do the right thing, they will get results.’ (Father, MM5, D3)

Despite this support, parents still reported some concern for their child's future.

‘it's quite hard, because I don't know what that's gonna hold for [child]…in the future.’ (Mother, MM3, D7).

### 2.10 Domain 3: Therapists and Communities

#### 2.10.1 Theme 3: Consistent, Knowledgeable and Caring Therapists

Therapists themselves were a large part of the strengths described by parents.

‘We've been extremely lucky, and I do often feel like we were given a magic wand…and I do not take that for granted in the slightest.’ (Mother, MM5, P2)

One parent reported that AHP's ‘have been so welcoming and nonjudgmental, and that's made my job [parenting and providing support for their child] so much…easier.’ (Father, MM5, D3)

They appreciated the AHP presence, but also their skills and ability to build relationships with their children.

‘I've been so lucky working with, and [OT] has been… absolute, absolute lifechanging…I couldn't imagine what it would be like if had we not had that support right from the beginning.’ (Mother, MM5, P2)

‘in themselves [AHPs]. They are a strength, you know…Their skill set and their ability to engage with children.’ (Mother, MM6, P1)

Parents reported that AHPs were able to ‘show us the next step ahead, I suppose, when we weren't able to see it, particularly in those early days.’ (Mother, MM5, D2) One parent broke down in tears when recounting how they felt during their experience accessing these services: ‘as a parent that goes through this…to be seen and heard was amazing.’ (Mother, MM5, D5)

A child also reported the value of keeping their therapists for a longer period of time:

‘I kind of like having them for longer, because then you can develop a better bond with them…after a certain amount of time, you kind of get to know each other pretty well, which makes conversations flow easier during your sessions.’ (15yo girl, MM5, C4)

These relationships families have with their therapists were seen to be specific to rural areas. ‘I don't feel like you would get those professional…those relationships and things in the city.’ (Mother, MM5, D2)

When asked what they would need to deliver their ‘Dream’ services, parents suggested, ‘having more incentives to keep people [AHPs] in the country for longer.’ (Mother, MM3, P5) These ‘Dream’ services often included ‘more’ access.

‘More consistency, more appointments, more connection.’ (Mother, MM5, D5)

Parents were aware of challenges faced by AHPs, with a parent sharing their experience working with an AHP struggling to find time for report writing.

‘she goes,'this is the time I've got to do my reports’ so, and that shows you how hard the system is.’ (Father, MM5, D3)

One parent suggested some support for AHPs admin work to help with ‘somebody to help them [AHPs] out with their paperwork as well.’ (Mother, MM3, D7)

#### 2.10.2 Theme 4: My Child and Their Therapist Are Both Parts of the Community

Parents discussed the importance of having their child as part of the community.

‘He's part of the community. He goes to the gym. He's at football. And I think in the rural communities that is super super important for our kids. It's their social life.’ (Mother, MM5, P4)

The value of being a part of community extended to wellbeing and also communities’ knowledge and understanding of disability.

‘I feel like that would draw people in too because who doesn't want to be part of a community?’ (Mother, MM5, D5)

Through being a part of the community, there was more understanding of their child and disability with a parent reported noticing other children in the community ‘[have] watched him grow up essentially.’ (Mother, MM5, D2)

Parents reported the importance of therapists being part of the community: ‘I suppose that accessing services locally, they know the challenges that you've that we face locally.’ (Mother, MM3, P5)

Being in smaller communities was a benefit reported by children because ‘country areas, they're normally smaller clinics. So, it makes it feel that little more personal.’ (15yo child, MM5, C4)

Parents reported the availability of local university AHP courses as something that was seen as having potential to increase the number of local AHP staff: ‘it is local students studying to do those things. So they're the ones that will be back into our area so that can only be a positive for all of us.’ (Mother, MM5, D2)

The impact of health services on the community was noted by parents/carers. This was reported as something parents would discuss amongst each other in social settings.

‘You go to coffee, and you're hearing 2 or 3 tables talking about the health services.’ (Mother, MM5, D5)

When asked about understanding the community services (such as allied health supports) available, parents were unsure of what was available.

‘I wouldn't know the first one to even suggest. Like yeah, got no idea really.’ (Mother, MM4, D1)

Parents ‘dreamt’ of having AHPs stay around and ‘to have the continuity of care that is accessible, because I feel like people out here are just forgotten (Mother, MM6, P6)’. This was particularly important for parents not to need to continuously tell their story.

‘not having to explain my child to every single clinician that I go to, it would just be heaven.’ (Mother, MM5, P4)

This also included having AHP staff collaborating and working with the same team.

‘all in the one like one team. So you don't have to keep explaining the story.’ (Mother, MM4, D1)

AHP's personal lives, and social factors, were noted to affect retention.

‘I guess, trying to make sure that they all just don't disappear and have babies.’ (Mother, MM6, P6)

Even where there were AHPs, parents reported staff were burdened.

‘at the moment we're so we're so lucky we have an OT that's here, as I said, but she's so overworked.’ (Mother, MM6, P6)

To keep AHP's around, parents ‘dreamt’ of having a stronger community presence, as in ‘[public health team] to be out in the community more. They need to be making a community presence.’ (Mother, MM5, D5) This could be achieved through building community offerings and physical spaces for new staff to come to.

‘you would need the people. It's that old saying, if you build it, they will come.’ (Mother, MM6, P1)

Finally, parents in one area reported that their local service had a poor reputation that they believed affected retention of staff and people's willingness to access health services.

‘I feel like to get people to come here…we'd need a better reputation [of health service].’ (Mother, MM5, D5)

### 2.11 Domain 4: Organisations/Services

#### 2.11.1 Theme 5: Flexible Environments and Health Services

Parents reported the benefits of flexible locations, in particular, home‐visit sessions.

‘having them come to us was a huge positive. And being able to see our kids in their natural environment, where they'd actually do all these things that we've been talking about.’ (Mother, MM6, P6)

Community hubs, putting AHP's all in one location, was ‘dreamt’ by parents.

‘make the services, probably. Yeah, all in one spot. But that would be about it. That's how simple it would be.’ (Father, MM5, D3)

This was reported to reduce time lost in children's days and the need for flexible sessions and wanting ‘therapists that come to us rather than losing hours per week in. Having to sign out of school early.’ (Mother, MM5, D4)

Where there was a challenge to accessing timely services, one parent reported the potential benefit of online services, such as the hospital's virtual care.

‘Can we have some help before our appointment or something? I feel there is a huge need, a huge gap in the market. Especially for people out here where we don't have you know, we're waiting months and months and months for services.’ (Mother, MM6, P6)

In many areas, there were limited services, resulting in reduced opportunities to access therapy that was needed. Or, in some instances, where parents were not entirely happy with a service, they reported having limited options.

‘It makes it hard when you know it's [going to] help. But you can't do it.’ (Mother, MM4, D1)

‘I guess our only option is to stay at that clinic.’ (Mother, MM5, D4)

Parents reported the inflexibility of services for certain ages, as often support for services will ‘time out’ at a particular age (e.g., at 7 years for some public health sectors).

‘the services…they're not always there, so like when [child] timed out at 7 [years old] from here. He didn't have therapy for a year, because we just couldn't get anyone.’ (Mother, MM5, D6)

### 2.12 Domain 5: Government

#### Theme 6: Accessing Funding Models and the Healthcare System

Parents/carers rarely described funding models and easy understanding of healthcare systems as a strength. More commonly these were positioned as areas they hoped to see improved in the ‘Dream’ stage. There was one example of a parent feeling like their funding model supported them when discussing their NDIS plans, stating, ‘they've been pretty good with what we've needed.’ (Mother, MM4, D1)

Funding models were reported as challenges to accessing services. This was extended to reasons for losing services due to the costs for service providers.

‘Maybe if the overheads weren't so high for the service providers and they might be able to offer a higher wage to the staff to give them an incentive to come work.’ (Mother, MM5, D4)

Some parents felt overwhelmed at the thought of making change to the health system: ‘that's the hard part…I just don't know how that would be possible’ (Mother, MM4, D1), whereas others wanted blanket changes: ‘I'd change everything about that system…Where do I start?’ (Mother, MM5, D4). This was at times reported due to challenges in navigating the system. Some were more circumspect.

‘A little bit easier to navigate the health system…It's hard to know where to even begin to access the services. And you really have to advocate for your child.’ (Mother, MM3, P5)

To achieve these ‘dream’ services it was suggested that having knowledge of what parents wanted was identified as way forward for improving systems:

‘Getting parents’ feedback. I think as well. It's always nice to be asked.’ (Mother, MM3, P5)

Additionally, having knowledge of the community was also reported as beneficial to improving the health system.

‘She had absolutely zero knowledge of the area. Every time I would ask her to put us in contact with different service providers, she would basically tell me to resort to a Google search.’ (Mother, MM5, D4)

Not having this understanding often presented further challenges for families. This was particularly relevant for those receiving services through the National Disability Insurance Scheme (NDIS); Australia's national funding scheme for those with a diagnosed disability.

‘you do these (NDIS) reports and you send that off and then…(NDIS plan writers) had no idea what was going on. And they've got no idea of your kids, circumstances…that side to it is hard.’ (Mother, MM5, D6)

## 3 Discussion

This study is among the first to explore the experiences of children and parents/carers in rural and remote areas accessing allied health services. Results from these interviews provide a strengths‐based understanding of the need for fun, family/child‐centred services that are connected to the community through knowledgeable and caring AHPs, to improve accessibility and outcomes. Framing the themes within the socio‐ecological framework provides insights into resources required to realise ‘Dream’ services, highlights what end‐users value, and empowers change. Ultimately, this study emphasises the importance of the relationship between families and their therapists, recognising both children's growth and the personal qualities of the therapist.

Parents/carers most consistently identified the primary strength of services as the AHPs themselves. Personal qualities such as knowledge, care and being community‐centred were highlighted. This aligns with existing literature on what makes an ‘excellent’ AHP, including fostering a sense of belonging and empowerment, along with opportunities for development [38]. However, this study is among the first to articulate this from the perspective of a health service user. Previous research has demonstrated the therapeutic value the child/family and interprofessional relationships hold in achieving positive health outcomes [39], and for our families, the value of the relationship could not be understated.

Rural and remote Australians have a deep attachment to their communities [40], and parents/carers in this study expressed the value they placed on their community, as well as the role of AHPs within it, particularly when therapists were local and demonstrated an understanding of the community context. Consequently, achieving a balance between professional responsibility and the development of positive relationships is especially important in rural and remote settings, also described in previous research [39]. There is additional evidence supporting the value of teams working together, particularly for complex care [41]. Parents/carers ‘dreamt’ of ‘community hubs’ as a way to improve accessibility by enabling therapists to deliver services directly within their towns. They also spoke of the significance of witnessing their child's growth throughout AHP sessions, attributing this to both the clinical skills of AHPs and the support provided to them in their parenting role. The importance of enhancing participation and community access for children with disabilities from a parental perspective has been highlighted in previous research [42] and is reflected in the International Classification of Functioning, Health and Disability (ICF) for children, the ‘F‐Words’ [43]. Our findings reinforce this, with parents speaking proudly of their child's ability to access their community, such as sporting clubs, following AHP intervention.

Healthcare access has been defined by Levesque et al [44] as comprising five stages: to identify needs, to seek services, to reach services, to use services and to have a need for services fulfilled. Parents/carers in this study reported challenges across multiple stages of healthcare access, particularly in seeking and reaching services, with poor experiences often leaving them feeling isolated and unsupported. Challenges were expressed across most stages of healthcare access, in particular, identifying needs – where to go for therapy (e.g., where do you go to get services started?), matching needs to therapists, working out next steps (e.g., developing goals) and accessing support for funding. Additional challenges were reported in ‘using services’, particularly navigating funding systems. In Australia, the NDIS has significantly influenced delivery of healthcare in the disability space since its introduction in 2013. This government scheme has increased people's financial ability to access vital supports for those with lifelong disabilities. However, administrative complexities within the scheme were frequently reported by parents/carers in this study. Notably, many participants described uncertainty at the earliest stages of access, including not knowing where to go or who to contact when first seeking services. There is persistent deficit‐based reporting of rural and remote peoples reduced healthcare seeking compared to metropolitan counterparts. The issues identified with healthcare access, reduced understanding of the community needs, and subsequent reduced healthcare seeking may impact communities willingness to engage with healthcare. Parents/carers suggested that greater community presence, such as visible physical service locations and locally based NDIS support coordinators, would improve their understanding of available services. The reputation of the local health services was also identified as an important facilitator of access. This is also furthered by research reflecting on the Institute of Medicine's domains of healthcare quality, which has suggested adding additional domains of ‘caring’ and ‘navigating the system’ [45]. Our research findings add support to the idea of these additional domains. These findings highlight the importance of local knowledge and contextual understanding in efforts to improve healthcare access. Healthcare access cannot be improved if the initial steps of supporting communities to recognise needs and understand how to seek services are overlooked.

While there are emerging examples of inclusion of children in qualitative research such as pain science [31], powered mobility interventions [46] and experiences of inclusion for autistic children [47], children have historically been excluded from research due to pragmatic reasons related to study design and methods [13]. This study differs by providing children living in rural and remote areas with the opportunity to use their voices to provide feedback on health service delivery. As children have their own experiences that may not be fully recognised or understood by adults in their lives, incorporating their views enriches understanding beyond what is reported by parents/carers and clinicians. Listening directly to children enabled insight into what is meaningful to them as the end‐users of services. Previous research with children with cerebral palsy has shown that children want to be spoken to more in medical consultations and that there are differences in the parents’ and children's experiences of care [48]. Results from our children's interviews further demonstrate that, for children, meaningful and ‘good’ therapy is fun and aligned with their interests. However, children predominantly responded to the initial question, providing data for Domain 1 only in the results. Other research has emphasised the importance of ‘fun’ in powered mobility interventions, with ‘play’ being a fundamental part of the intervention [46]. ‘Play’ is widely understood as a child's occupation [49], underscoring the need for its intentional inclusion within therapy sessions to support participation and therapeutic benefit. Additionally, one of the ICF ‘F‐Words’ is ‘fun’ and considers the ‘doing’ and being involved with ‘fun’ activities, as meaningful participation for children [43]. Our findings extend this evidence by demonstrating that fun is not only therapeutically valuable but also intrinsically valued by children.

This research has many implications for future service design in rural and remote areas. Elements from our research highlighted the strengths/resources of ensuring fun sessions, using local and caring clinicians, and supported health service access. The focus on South Australia provides us with context‐specific information that tells us what is valued and seen as a strength in current services. There is increasing research on the value of place‐based approaches [50]. These approaches are considered to have elements such as strong partnership, valuing local knowledge and having community accountability [51]. Through this place‐based lens, and a strength‐based lens we can look at broader and different resources to what might been seen in a ‘deficit‐based’ approach to build improved services for children in rural and remote areas. The inclusion of children, and inclusive methods to do so, are a strength to this paper. A result of this has meant the voices of children with various diagnoses in rural and remote South Australia have been able to have their voices heard. The use of a strengths‐based approach has the ability to shift the deficit‐based understanding of rural health service delivery and instead allow us to see what strengths we may look for when trying to impart positive change. Limitations to this study include a lack of representation from some areas of rural and remote Australia, due to challenges with recruitment. Additionally, the parents recruited were those who were engaged in the service. As a result, this research was unable to capture those who had disengaged from services due to snowball sampling technique and the challenges related to contacting ‘unable‐to‐contact’ families. While we judged the resulting sample to hold adequate information power (see Methods), we acknowledge that a larger or differently recruited sample might have surfaced additional perspectives. Additionally, children's voices were concentrated to Domain 1, likely reflecting the need to provide more structured questioning required to achieve detail in answers to more complex questions. Future research should consider alternative developmentally adapted method (e.g., shorter formats) to more fully capture child perspectives.

## 4 Conclusion

Children and parents/carers in rural and remote South Australia described fun, engaging sessions with knowledgeable and caring AHPs as core strengths of their local services. Children's voices offered unique insights into what makes therapy meaningful, while parents/carers emphasised the value of relationships with AHPs and the importance of guidance to navigate complex healthcare systems. The value of ‘fun’ and community cannot be understated in delivering strong allied health services. Bringing these strengths together with end‐users ‘Dream’ ideas within the socio‐ecological framework creates a clear path for action. When children's perspectives are centred and families are supported to understand the system around them, communities are positioned to drive sustainable and practical improvements in allied health service delivery.

## 5 Author Contributions

**Georgia Gosse:** conceptualisation, methodology, writing – review and editing, writing – original draft, data curation, investigation, formal analysis, project administration. **Saravana Kumar:** supervision, conceptualisation, methodology, writing – review and editing, resources, investigation. **Helen Banwell:** supervision, conceptualisation, methodology, writing – review and editing, resources, investigation. **Joshua Pate:** supervision, writing – review and editing, methodology, investigation.

## 7 Ethics Statement

This project was approved by the Women's and Children's Network Human Research Ethics Committee (HREC) (Reference: 2024HRE0180) and the University of South Australia HREC (Reference: 206707). Site‐specific assessments were gained from all six regional local health networks in SA. All participants provided written consent to participate in this study.

## 8 Conflicts of Interest

The authors declare no conflicts of interest.

## Supporting information


Supporting File 1

Supporting File 2

Supporting File 3

## Data Availability

The data that support the findings of this study are available from the corresponding author upon reasonable request. Additional data related to this study are available from the corresponding author upon reasonable request.
