# Supplementary material for: ‘I Do Often Feel Like We Were Given a Magic Wand’ – Children's and Families’ Experiences of Allied Health Services for Developmental and Disability Needs in Rural and/or Remote South Australia
Source: Health Expect. 2026 Aug 14;29(4):e70820. doi: 10.1111/hex.70820 (PMC13474004; doi:10.1111/hex.70820)
Supplement: Supplementary file 2 [file HEX-29-e70820-s001.docx]

**BACKGROUND INFO – QUESTOINNAIRE.**

*This was developed into a Microsoft form for convenience when filling out. This will be completed with the presence of the lead researcher so that the interviewee is given the opportunity to ask questions.*

**PARENTS/CARERS**

1. Gender

☐Male

☐Female

☐Non-binary

☐Prefer not to say

2. DOB: DD/MM/YYYY
3. Postcode:

___________________________________________________________________________

4. Do you identify as Aboriginal and Torres Strait Islander?

☐Yes – Aboriginal

☐Yes - Torres Strait Islander

☐Yes - Aboriginal and Torres Strait Islander

☐No

☐Prefer not to say

5. Do you identify as culturally and linguistically diverse?

☐Yes

☐No

If yes, please specify languages other than English spoken: ___________________________

6. How many children (under the age of 18) currently live with you?:

☐1

☐2

☐3

☐4

☐5

☐6

☐7

☐8

☐9

☐10

7. How many of these children have received allied health support previously?:

☐1

☐2

☐3

☐4

☐5

☐6

☐7

☐8

☐9

☐10

8. How many of these children still receive allied health support?:

☐1

☐2

☐3

☐4

☐5

☐6

☐7

☐8

☐9

☐10

9. How long have you been accessing (or accessed if discharged in the last 6 months) the allied health service:

________________________________________________________________________

10. Most recently, did you receive allied health services in public or private setting?:

☐ Public

☐ Private

**skip logic from question 8 so that 11, 12 and 13 are organised for each children (i.e., if 4 children, these will repeat 4x).

11. Allied health services seen within the last 12 months (leave blank for a child recently discharged). Select all that apply:

Choose an item.

** Disciplines in drop-down – Audiologist, Dietician/Nutritionist, Exercise Physiologist, Physiotherapist, Podiatrist, Psychologist, Social Worker, Speech Pathologist

12. Age of child/ren currently receiving services: _______________________________________________
13. Does your child/ren have an existing medical condition or syndrome that is related to them seeing this service?:

_______________________________________________________________________

14. Context in how services are received (select all that apply):

☐Clinic based

☐Visiting clinics

☐Home visits

☐School visits

☐Teletherapy

Other:__________________

15. Model of Care (as per Gosse 2024 reference)* (select one or more):

☐Screening service

☐Consultative service

☐Role substitution

☐Online service

☐Typical clinic-style service

Other: ___________________

**Note** – the below descriptors will feature at the top of the question when developed into an online form.

**Definitions of models of care - Screening service: a service delivered with the intention of identifying children with needs and referring to relevant services; Consultative service: a service that aims to educate other allied health professionals of members of the team around the child (parents/carers, education staff etc); Role substitution: a transdisciplinary style service with an AHP takes on a role outside of typical role of their discipline; Online service: any service that uses technology such as teletherapy or online websites; Typical clinic style service: services delivered within the clinic as a typical style of delivery.*

*Other – if you do not believe your service delivery style is adequately covered by any of the above*

**CHILDREN**

***this questionnaire will be filled in with support from parent/carer with lead researcher nearby.*

1. Gender

☐Male

☐Female

☐Non-binary

☐Prefer not to say

16. DOB: DD/MM/YYYY
2. Do you identify as Aboriginal and Torres Strait Islander?

☐Yes – Aboriginal

☐Yes - Torres Strait Islander

☐Yes - Aboriginal and Torres Strait Islander

☐No

☐Prefer not to say

3. Do you identify as culturally and linguistically diverse?

☐Yes

☐No

If yes, please specify languages other than English spoken: ___________________________

4. Diagnosis (if any): ______________________________________________________
5. Most recently, did you receive allied health services in public or private setting?:

☐ Public

☐ Private

6. Allied health services seen (including services no longer seen). Select all that apply:

Choose an item.

** Disciplines in drop down – Audiologist, Dietician/Nutritionist, Exercise Physiologist, Physiotherapist, Podiatrist, Psychologist, Social Worker, Speech Pathologist

7. Currently seeing allied health services:

☐Yes

☐No

8. Allied health services currently seen. Select all that apply (of leave blank if discharged from services in last 6-12months):

Choose an item.

** Disciplines in drop down – Audiologist, Dietician/Nutritionist, Exercise Physiologist, Physiotherapist, Podiatrist, Psychologist, Social Worker, Speech Pathologist

9. Where do you see/previously saw your therapist (select all that apply)?:

☐Clinic based

☐Visiting clinics

☐Home visits

☐School visits

☐Teletherapy

Other:__________________
