# Supplementary material for: ‘I Do Often Feel Like We Were Given a Magic Wand’ – Children's and Families’ Experiences of Allied Health Services for Developmental and Disability Needs in Rural and/or Remote South Australia
Source: Health Expect. 2026 Aug 14;29(4):e70820. doi: 10.1111/hex.70820 (PMC13474004; doi:10.1111/hex.70820)
Supplement: Supplementary file 3 [file HEX-29-e70820-s002.docx]

**INTERVIEW QUESTIONS**

**PARENTS/CARERS**

Discovery

Thinking about the allied health service you receive/d for children, can you share a good experience?

Dream

If you could wave a magic wand, and you could have anything you want, what would these allied health services look like?

Design

If you had a friend who needed these services, what sort of things could make sure it was the best experience for them?

Destiny

Looking into the future, how can we make sure these changes will last?

**Introduction:**

- Lead researcher/interviewer to introduce herself and thank the participant and remind participant they can stop at any time.
- Lead researcher/interviewer to introduce additional researcher who will be on an online call recording and taking notes. The second researcher will not have video enabled on screen.

**Discovery:**

- Thinking about the allied health service you have receive/d for your child, can you share a positive experience?

*If the participant is struggling to report a time:*

- Was there a time where you felt particularly supported, heard or empowered?
- Or move on to questions 2 and 3 for this section.

*If the participant reports a time and you need more detail on the service delivery:*

- What do you think contributed to that experience?
- Is there anything in the way that service is delivered that made that possible?

*If the interviewer does not understand a situation or experience described by the participant then ask…*

- Help me to understand a little better what you meant when you spoke about [insert scenario/statement/etc that is unclear to the researcher].

*If not covered in follow up questions – continue with:*

- If you were going to tell you friend about your experience with [this allied health service] if they needed for their child, what would you tell them?
- What are some things that you see as strengths?
- Are there any other things you like about the allied health service you access for your child?

*Follow-up questions if they do not understand or do not provide enough detail:*

- What parts of the service do you think work the best? (if really struggling can list elements including referrals, initial appointments, follow-ups and collaboration with other people or therapists in your child’s life)
- Tell me more about [insert element that they have discussed that you want to know more about].

**Dream:**

- If you could wave a magic wand, and you could have anything you want, what would allied health services for your child look like?

*If the response lacks some detail about the specific allied health service :*

- How can allied health services contribute to the overall well-being of children and families in rural areas?

*If details on ideal outcomes are omitted, ask:*

- What outcomes would you hope to achieve by making these services?

*If the participant is unsure with what to say, then ask:*

- What are some changes you would like to see?
- What would the best service for kids like your child in the country look like?

**Design:**

- If you had a friend who needed these services, what sort of things could make sure it was the best experience for them?

*This section is more strategy focused so could prompt in this direction.*

*If the participant mentions some elements in their dream phase that are not covered here, follow up with:*

- How can we achieve [insert element they discussed in dream phase e.g., collaboration, different contexts]

**Destiny**

- Looking into the future, how can we make sure these changes will last?

*This question may have limited detail due to some similaries from previous questions. Due to there being nothing implemented in this project, it can be less detailed. However, if they are stuck for ideas, there are some prompting elements that could be raised:*

- What proactive steps could you personally take?
- Other organisations or groups of people that could help?

**Closing:**

- That is the end of my questions. Is there anything that you would like to add or you feel like has not been covered?
- A reminder that for your participation you will receive at $25 Coles/Myer group voucher.
- Ask for mailing address if seen virtually.

**CHILDREN**

Discovery

Thinking about [the allied health service] you receive with [name of allied health professional], can you share a good experience?

Dream

If you could wave a magic wand, and you could have anything you want, what would these [allied health] services look like?

Design

If your [friend, toy, superhero etc] was going to come in and receive services, what could we do make it the best experience for them?

Destiny

Looking into the future, how can we make sure these changes will last?

**Room set-up:***

*Dependent on what is available in the clinic environment used for interviews.

- Drawing items
- Dolls
- Dinosaurs/artefacts

**Introduction:**

- Lead researcher/interviewer to introduce herself and thank the participant and remind participant they can stop at any time.
- Lead researcher/interviewer to introduce additional researcher who will be on an online call recording and taking notes. The second researcher will not have video enabled on screen.
- Lead researcher/interviewer to ask child what sort of things they like to play with and start to set up some play for discussion to start.

**Discovery:**

- Thinking about [the allied health service] you receive with [name of allied health professional], can you share a good experience?

*If the participant is having difficulty reporting an experience:*

- Was there a time where you felt particularly supported or empowered?
- Or move on to questions 2 and 3 for this section.

*If the participant continues to have difficulty reporting an experience, they can be offered to draw or to play. Once the drawing or play has been established, interview could use the following questions:*

- Tell me more about what this means? [indicate part of drawing or play set up]
- Why have you chosen [colour pencil, type of toy etc]

*If the participant reports a time and you need more detail on the service delivery:*

- What do you think made that so good?
- Is there anything in the way that you have [allied health service] that made that possible?

*If the interviewer does not understand a situation or experience described by the participant then ask…*

- Help me to understand a little better what you meant when you spoke about [insert scenario/statement/etc that is unclear to the researcher].

*If not covered in follow up questions – continue with:*

- What are some things that make this good?
- Are there any other things you like about your service?
- What would you tell one of your friends about the service?

*Follow-up questions if they do not understand or do not provide enough detail:*

- What parts of the service do you think work the best? (if really struggling can list elements including referrals, initial appointments, follow-ups and collaboration with other people or therapists in your child’s life)
- Tell me more about [insert element that they have discussed that you want to know more about].

*If the participant reports feeling unclear of what kind of positive experience we are referring to, then ask…*

- Was there a time when you felt particularly successful in supporting your families?

**Dream:**

- If you could wave a magic wand, and you could have anything you want, what would these [allied health] services look like?

*If the response lacks some detail about the specific allied health service :*

- What would the best service for kids like you in the country look like?

*If the participant continues to have difficulty reporting, they can be offered to draw or to play. Once the drawing or play has been established, interview could use the following questions:*

- Tell me more about what this means? [indicate part of drawing or play set up]
- Why have you chosen [colour pencil, type of toy etc]

*If details on ideal outcomes are omitted, ask:*

- What outcomes would you hope to achieve by making these services?

*If the participant is unsure with what to say, then ask:*

- Are there some changes you would like to see to your [allied health service they see]?

**Design:**

If your [friend, toy, superhero etc] was going to come in and receive services, what could we do make it the best experience for them?

*If the participant mentions some elements in their dream phase that are not covered here, follow up with:*

- How can we achieve [insert element they discussed in dream phase e.g., collaboration, different contexts]

*If the participant continues to have difficulty reporting, they can be offered to draw or to play. Once the drawing or play has been established, interview could use the following questions:*

- Tell me more about what this means? [indicate part of drawing or play set up]
- Why have you chosen [colour pencil, type of toy etc]

**Destiny**

What could we do to make sure [changes they suggested] happen and last?

*This question may have limited detail due to some similaries from previous questions. Due to there being nothing implemented in this project, it can be less detailed. This could also be challenging for younger children.*

Some potential prompts could include:

- What could kids like you do?
- Are there any other groups of people who could help to make sure this happens?

*If the participant continues to have difficulty reporting, they can be offered to draw or to play. Once the drawing or play has been established, interview could use the following questions:*

- Tell me more about what this means? [indicate part of drawing or play set up]
- Why have you chosen [colour pencil, type of toy etc]

**Closing**

- That’s all the questions I have! Is there anything else about your [allied health] therapy sessions that you want to tell me about?
- Thanks so much for joining me. I have a voucher you can use at the shops to say thanks for helping out.
- Ask parent/carer for mailing address if seen virtually.
